# Queer Things about Mankind

**Published:** 1898-02

**Authors:** 


ARTICLE VIII.

### QUEER THINGS ABOUT MANKIND.

Few people are aware of the wonderful engineering skill and ingenuity with which their bodies are constructed. If patents were taken out for all the clever contrivances to be found there, it would probably keep the staff of the Patent Office going for three months.

Who would think that in his eye there is a block and pulley, or "tackle" as the sailor calls it, as complete and efficient as that with which a ship hoists her mainsail? There it is, however, and whenever you look at the tip of your nose the muscle that moves your eyeball works in it. There are several of these pulleys in the body.

Another clever dodge in Nature is shown in ths bones of the face. Accomplished engineer that she is, she always uses the smallest quantity of material sufficient for strength. In making the bones of the face she wanted a large surface to which to attach the muscles; but as she didn't wish to encumber us with heads as heavy as an elephant's, she burrowed hundreds of little holes in the bones, called air-cells, and thus secured strength, large surface and lightness. In the same way she made the long bones of the legs and arms hollow in the middle. What a saving this is may be understood from the fact that a hollow shaft of bone or iron—or any other substance—is about twice as strong as a solid shaft containing the same quantity of material.

When you get a severe cold you are apprised of the presence of another conning device—the Eustachian tube. This tube is two inches long and passes from the inside of the ear to the back of the mouth. It was put there to keep the air at the same pressure inside the drum as outside. Otherwise there would be no vibration of the drum and you would be almost stone deaf. When you get a bad cold this tube sometimes becomes inflamed and blocked, and you are made quite deaf.

Adam's apple, if it was that fruit which brought into the world all our woe, is now a useful organ. It serves as a sort of storage cistern of the blood for the supply of the brain. When the heart sends up too much blood Adam's apple intercepts it, or part of it; and when the direct supply from the heart temporarily runs short Adam's apple gives up its store.

The liver is a most wonderful organ, containing facilities of several kinds. But perhaps the most wonderful thing in it is that part set aside to look for and arrest poisons.

All the food that you eat, except the fat, has to pass through the liver before going to the heart and body generally; and in the liver there appears to be stationed something in the nature of custom officers, who examine every bit of food and remove from it all substances dangerous to the body. But they are capable of dealing only with the small quantities in ordinary food and when you eat poisonous mushrooms or mussels they are quite overpowered.

Another protection from danger is afforded you by the supply of a small quantity of hydrochloric acid to the stomach. There are little machines in the stomach especially designed for the manufacture of this acid from the salt you eat, and they are so regulated that they produce a quantity equal to one-fifth of one per cent of the contents of the stomach. Experiment shows that this is exactly the percentage required to destroy the thousands of microbes that we swallow in our food. But for this thoughtful provision of Nature we would probably get a new disease with every meal.

Most people know the use ot the epiglottis, which saves us from imminent death every time we swallow a bit of food. At the back of the mouth the air-passage and food-passage cross each other, and whenever we swallow food it would inevitably go into the wind-pipe and choke us; only that this little body pops down and covers the entrance. It is like the policeman who regulates the traffic where streets cross.

The semicircular canals, for centuries a physiological puzzle, are an extraordinary device for enabling us to keep our balance. They are like channels, hollowed out, in connection with the ear, in the bones of the head, and partly filled with fluid lymph. As our head or body sways the head moves, acting like a spirit-level, and informing the brain whether we are standing on the perpendicular or at a dangerous angle.

One of the most valuable of all inventions made for our comfort and safety is the perspirative gland. It acts like the safety valve of a boiler, letting off heat when we are becoming dangerously warm. If our temperature rose seven or eight degrees we should not have twenty-four hours to live. The value of the sweat-gland is therefore obvious. In fact, without it a football or cricket or rowing match would be out of the question, and we could not safely walk at a speed of more than a quarter of a mile an hour. Nature has taken good care, however, that we should not run short of these useful organs and has given us no less than 2,500,000 of them.

So inventive was Nature when constructing our body that the difficulty is to stop enumerating her clever ideas. She saw that we would very soon grow tired if we had to be held up by two legs by means of muscular effort, so she made the hip-joint air-tight, and the pressure of the air alone keeps the leg in its place. At the same time, although she has not discovered ball-bearings, she made the ball of the leg bone and the socket of the hip so smooth and oiled the joint so well that the friction is practically nothing.

When the spinal canal in the backbone was made, great pains had to be taken, for, while it consists of many pieces and is freely movable, it contains the precious cord, one nip of which would be fatal. The measurements are so accurate that there is no danger of such an event. Whenever there is much and free motion, as in the neck, the canal is large and open and nip is impossible.

Again, the heart and lungs are, of course, the very basis of our life. They are in constant motion, and if allowed to rub against the cheft walls around them, they would either get inflamed or wear away by friction. Nature has, therefore, surrounded them with a double sac, and between the outer and inner layers of it she has placed a quantity of lubricating fluid.

But the most remarkable of all devices is that for splicing broken bones. The moment a bone is broken a surgical genius is at once dispatched from the brain to the spot. He proceeds to surround the broken ends with a ferule of cartilage. This is large and strong, and takes quite a month to complete. When the two ends are held firmly and immovably in place by the ferule, this mysterious surgeon begins to place a layer of bone between them and solder them together. And when the layer is complete and the bone securely welded he removes the ferule or callus, just as the scaffolding is removed from a finished building. Often a bone does not get broken for two or three generations, and yet this power to form the callus and knowledge of how to do it is never lost.—*Scientific American.*

